# Metabolic GWAS of elite athletes reveals novel genetically-influenced metabolites associated with athletic performance

**DOI:** 10.1038/s41598-019-56496-7

**Published:** 2019-12-27

**Authors:** Fatima Al-Khelaifi, Ilhame Diboun, Francesco Donati, Francesco Botrè, David Abraham, Aroon Hingorani, Omar Albagha, Costas Georgakopoulos, Karsten Suhre, Noha A. Yousri, Mohamed A. Elrayess

**Affiliations:** 1 Anti Doping Laboratory Qatar, Sports City, Doha, Qatar; 2 Division of Medicine, University College London, London, NW3 2PF United Kingdom; 3 College of Health and Life Sciences, Hamad Bin Khalifa University, Doha, Qatar; 4 Laboratorio Antidoping, Federazione Medico Sportiva Italiana, Largo Giulio Onesti 1, 00197 Rome, Italy; 5 UCL Institute of Cardiovascular Science, University College London, London, WC1E 6BT United Kingdom; 6 Center for Genomic and Experimental Medicine, University of Edinburgh, Edinburgh, UK; 7 Department of Physiology and Biophysics, Weill Cornell Medical College in Qatar, Qatar-Foundation, P.O. Box 24144, Doha, Qatar; 8 Department of Genetic Medicine, Weill Cornell Medical College in Qatar, Qatar-Foundation, P.O. Box 24144, Doha, Qatar; 9 Computer and Systems Engineering, Alexandria University, Alexandria, Egypt; 10 Biomedical Research Center, Qatar University, Doha, Qatar

**Keywords:** Genome-wide association studies, Predictive markers

## Abstract

Genetic research of elite athletic performance has been hindered by the complex phenotype and the relatively small effect size of the identified genetic variants. The aims of this study were to identify genetic predisposition to elite athletic performance by investigating genetically-influenced metabolites that discriminate elite athletes from non-elite athletes and to identify those associated with endurance sports. By conducting a genome wide association study with high-resolution metabolomics profiling in 490 elite athletes, common variant metabolic quantitative trait loci (mQTLs) were identified and compared with previously identified mQTLs in non-elite athletes. Among the identified mQTLs, those associated with endurance metabolites were determined. Two novel genetic loci in FOLH1 and VNN1 are reported in association with N-acetyl-aspartyl-glutamate and Linoleoyl ethanolamide, respectively. When focusing on endurance metabolites, one novel mQTL linking androstenediol (3alpha, 17alpha) monosulfate and SULT2A1 was identified. Potential interactions between the novel identified mQTLs and exercise are highlighted. This is the first report of common variant mQTLs linked to elite athletic performance and endurance sports with potential applications in biomarker discovery in elite athletic candidates, non-conventional anti-doping analytical approaches and therapeutic strategies.

## Introduction

The superior physical performance of elite athletes is a multifactorial trait, with contributions from both environmental (exercise and diet) and genetic factors^1^. There is ample evidence suggesting influence of multiple genetic variants with small effect size over several phenotypic traits related to physical performance^2^. The identification of these variants is crucial to understand the superior performance of elite athletes and has been a subject of study for many years^3–5^. However, research into the genetics of athletic performance has been hindered by small sample sizes and complexity of the phenotype^6^. Genome-wide association studies (GWAS) in athletes versus non-athletes have uncovered many new loci^7,8^. However, a meta-analysis of 1520 endurance athletes and 2760 controls has revealed no evidence of association of a common genetic variation with endurance status in world class athletes^9^.

The advancement in metabolomics tools including mass spectrometry (MS) technologies has offered a unique opportunity to complement genomics data with intermediate phenotypes. Identified metabolites exhibited direct functional associations with genetic variants and provided greater effect sizes^10,11^. In a pilot metabolomics study, we identified differences in metabolic profiles between moderate and high endurance elite athletes including metabolites involved in steroid biosynthesis, fatty acid metabolism, oxidative stress and energy-related molecular pathways^12^. The integration of genomics and metabolomics technologies has also allowed a more comprehensive coverage of the metabolic pathways involved in complex physiological and pathological processes^13,14^.

GWAS for metabolic traits (mGWAS)^10,15–24^ has revealed hundreds of metabolomics quantitative trait loci (mQTLs) in the general population^22–27^. The identification of novel mQTLs in athletes who experience unique environmental conditions including special diet and intensive exercise may provide invaluable tools for biomarker discovery in relation to exercise and performance. This unique approach could provide better informed selection of athletic candidates and crucial information needed for optimal balance between training and recovery for every athlete^12^. Identified mQTLs in elite athletes could also help in the development of non-conventional anti-doping analytical strategies by understanding the genetic predisposition of specific doping-related metabolites. Furthermore, elite-athletes-unique mQTLs could offer potential novel therapeutic targets in athletes and potentially general population.

The aims of this study are (1) to confirm previously published^12^ metabolites associated with endurance sports, (2) to discover novel genetic loci affecting metabolites in elite athletes by fine-mapping loci to putative functional variants at or near sentinel SNPs (a sentinel SNP or sentinel metabolite refers to a lead SNP or a lead metabolite) and (3) to discover novel variant loci associated with endurance metabolites underscoring the metabolic individuality of endurance athletes.

## Results

**Figure 1:**
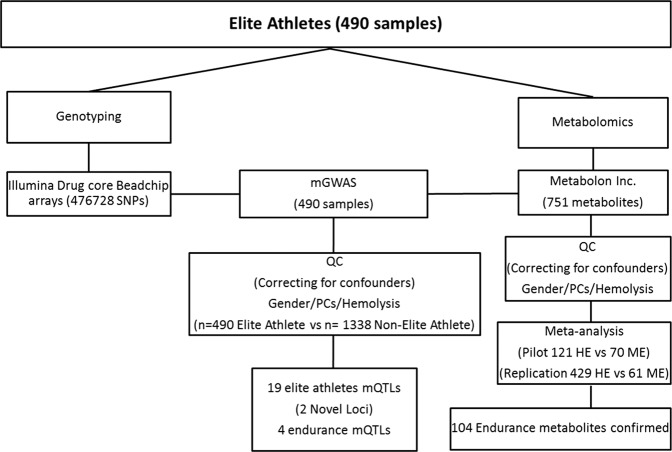
Schematic view of the study design. HE: High Endurance, ME: Moderate Endurance, QC: Quality Control, PCs: Principle Components.

Genotyping of 275,016 SNPs that passed quality control measures (see methods) was performed in 490 elite athletes belonging to different sport disciplines (Table S1), followed by serum metabolomics of 751 metabolites to confirm previously published endurance metabolites^12^. Subsequent mGWAS analysis was performed to reveal novel SNP-metabolite associations by comparing mGWAS hits identified in elite athletes with reference studies that were previously performed in non-elite athletes^25–27^. Finally, novel mGWAS hits associated with endurance sports were determined. Figure 1 provides a schematic representation of the study design.

### Confirmation of endurance-associated metabolites in elite athletes

In order to confirm previously reported associations^12^, a linear model was used to assess the significance of metabolite associations with the endurance level of athletes’ sports (moderate vs high endurance) after correcting for gender, hemolysis levels, PCA (PC1 and PC2 from metabolites) and ethnicity groups in a second cohort of 490 athletes. A meta-analysis confirmed 104 metabolites identified in both cohorts in association with endurance sports (Table S2), including elevation in pregnenolone, androgenic steroids and monohydroxy fatty acids and reduction in diacylglycerols, acyl carnitines, gamma glutamyl amino acids and glutathione in the high endurance sports.

### Common variant loci influence metabolites (mQTLs) in elite athletes

**Figure 2:**
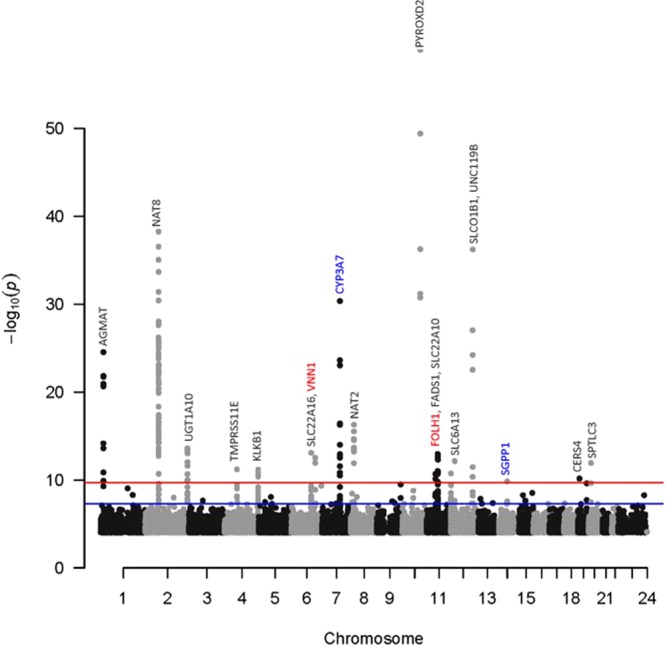
Manhattan plot for the discovered mGWAS loci. The red line indicates the Bonferroni threshold (2.4 × 10^−10^) and the blue line indicates the genome wide significance threshold (5 × 10^−8^). The novel gene/metabolite associations appear in red and the known gene/metabolite associations, but with novel SNPs, appear in blue. Previously reported associations are shown in grey.

**Figure 3:**
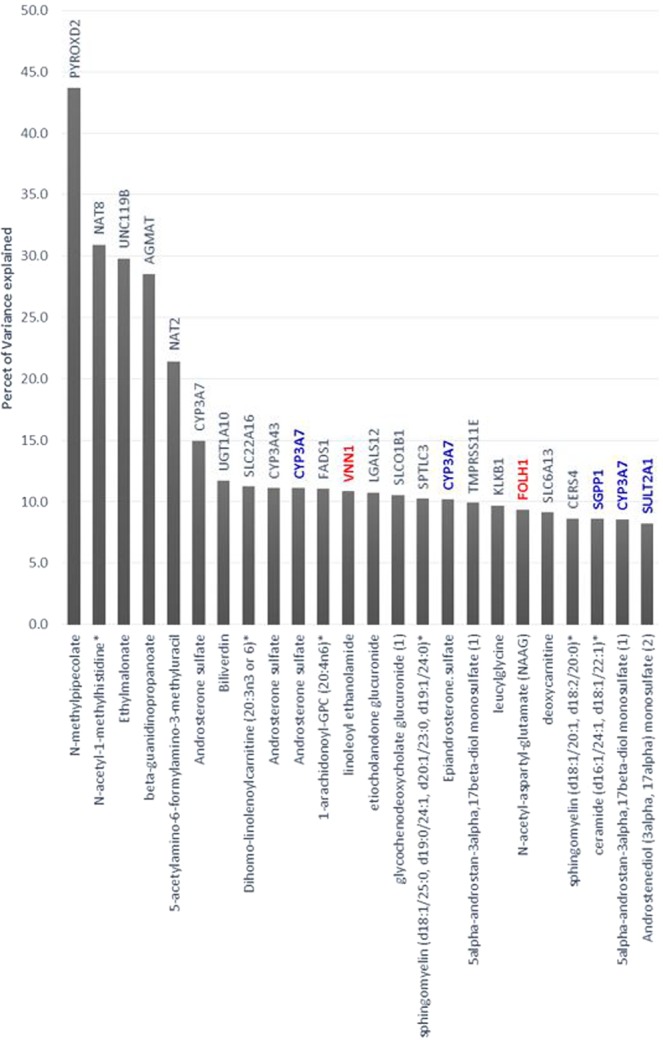
Percent of explained variance of metabolite by the corresponding SNP in the identified mGWAS loci in elite athletes. The height of a column bar reflects the percent of variance explained for each locus. Loci genes are indicated above the column bar and corresponding metabolite name on the X-axis. Novel mGWAS loci appear in red and previously reported associations are shown in in black. The known replicated loci, but with novel SNP or metabolite are typed in blue. Bars are colored according to Metabolon specified pathway for the metabolites associated with the locus. Biochemical Name* indicates compounds that have not been officially confirmed based on a standard, but Metabolon is confident in their identities.

By combining genotyping and metabolomics data, 145 significant SNP-metabolite associations (Bonferroni p ≤ 2.4 × 10^−10^) were identified (Table S3), with an average inflation factor for mGWAS metabolites of 1.07 (0.96–1.19). Genetic loci were investigated for known expression quantitative trait loci (eQTLs), mQTLs and functional associations using several databases including SNIPA http://snipa.helmholtzmuenchen.de/snipa/, PhenoScanner V2 A database of human genotype-phenotype associations http://www.phenoscanner.medschl.cam.ac.uk/, GTEx portal (version 2.1, Build #201) www.gtexportal.org, OMIM www.omim.org, Overview of Bravo variant server resources https://bravo.sph.umich.edu/freeze3a/hg19/ and GnomAD http://gnomad.broadinstitute.org/. By identifying the identities of their genes, these associations collapsed into 19 independent loci (Table 1, Fig. 2). The variance explained by these SNPs ranges from the highest value of 43.68% (N-methylpipecolate with rs7072216 in PYROXD2 locus) to the lowest value of 8.59% (Ceramide-d16:1/24:1-d18:1/22:1 in SGPP1 locus) with an average of 16.09% (Fig. 3).

**Table 1:** Nineteen unique locus-metabolite mGWAS pairs identified in 490 elite athletes, including two novel gene/metabolite associations and four known gene/metabolite associations but with novel SNPs.

Elite athletes										Non-elite athletes^27^			Non-elite athletes^25^		
Locus	rs ID	MAF	Metabolite	P value	Beta	SE. Beta	r2 (%)	Function (GVS)	Comment	Beta	GWAS p-value	r2 (%)	Beta	Locus p-value	r2 (%)
FOLH1	rs55729124	0.06	N-acetyl-aspartyl-glutamate (NAAG)	2.17E-11	−0.95	0.14	9.35	Intron	Novel gene/metabolite association						
VNN1	rs3798793	0.42	Linoleoyl ethanolamide	3.15E-13	0.46	0.06	10.82	Intron							
SGPP1	rs17101394	0.17	Ceramide (d16:1/24:1, d18:1/22:1)*	1.52E-10	0.52	0.08	8.59	Intergenic	Reported SNP association but with different metabolites	0.38	3.76E-16	2.4			
CYP3A7	rs11568825	0.01	Androsterone sulfate	3.91E-17	−1.88	0.21	14.11	Upstream-gene							
		0.01	Epiandrosterone sulfate	2.82E-12	−1.55	0.22	9.95	Upstream-gene							
		0.01	5alpha-androstan-3alpha,17beta-diol monosulfate (1)	3.31E-11	−1.53	0.23	9.2	Upstream-gene							
CYP3A7	rs45446698	0.03	Androsterone sulfate	4.62E-31	−1.92	0.15	24.82	Upstream-gene	Reported	−0.13	1.02E-126	0.5			
AGMAT	rs6429759	0.48	Beta-guanidinopropanoate	2.98E-25	0.74	0.07	28.54	Intron		0.28	1.57E-14	2.3			
CERS4	rs7258249	0.46	Sphingomyelin (d18:1/20:1, d18:2/20:0)*	7.17E-11	0.40	0.06	8.6	Upstream-gene		−0.3	5.02E-16	2.6			
FADS1	rs174547	0.30	1-arachidonoyl-GPC (20:4n6)*	1.18E-13	−0.48	0.06	11.04	Intron		−0.62	7.86E-69	10.2			
KLKB1	rs3733402	0.48	Leucylglycine	6.80E-12	0.40	0.06	9.68	Missense		−0.54	2.84E-53	8.8			
NAT2	rs1495741	0.27	5-acetylamino-6-formylamino-3-methyluracil	5.48E-17	0.74	0.08	21.44	Intergenic		0.57	1.39E-134	4.9			
NAT8	rs1881245	0.27	N-acetyl-1-methylhistidine*	5.76E-39	0.87	0.06	30.92	Intron		0.28	1.85E-93	1.8	−0.78	4.4 E-47	26.6
PYROXD2	rs7072216	0.35	N-methylpipecolate	1.23E-59	−0.96	0.05	43.68	Intron					−0.66	9.13 E-26	18.3
SLC22A10	rs75859219	0.06	Etiocholanolone glucuronide	5.04E-13	0.96	0.13	10.73	Upstream-gene		0.86	4.18E-35	4.8			
SLC22A16	rs12210538	0.24	Dihomo-linolenoylcarnitine (20:3n3 or 6)*	8.67E-14	−0.54	0.07	11.24	Missense		−0.42	3.27E-24	3.8			
SLC6A13	rs11613331	0.46	Deoxycarnitine	1.93E-11	0.39	0.06	9.15	Intron		−0.44	4.26E-40	5.8			
SLCO1B1	rs4363657	0.14	Glycochenodeoxycholate glucuronide (1)	7.18E-13	0.69	0.09	10.53	Intron		−0.24	7.74E-37	0.9	0.83	3.06 E-31	18.5
SPTLC3	rs680379	0.35	Sphingomyelin (d18:1/25:0, d19:0/24:1, d20:1/23:0, d19:1/24:0)*	1.20E-12	0.49	0.07	10.24	Intergenic		0.24	7.91E-09	1.5			
TMPRSS11E	rs35307342	0.36	5alpha-androstan-3alpha,17beta-diol monosulfate (1)	6.48E-12	0.47	0.07	9.9	Intron		−0.54	2.14E-07	1.1	−0.74	3.28 E-35	21.4
UGT1A10	rs10168416	0.31	Biliverdin	2.67E-14	0.50	0.06	11.68	Intron		−0.27	7.81E-70	0.5			
UNC119B	rs2066938	0.29	Ehylmalonate	6.16E-37	0.82	0.06	29.79	3-prime-UTR		0.96	1.11E-299	1.5			

r2 is percent of explained variance. Highlighted rows indicate novel significant mGWAS. Biochemical Name* indicates compounds that have not been confirmed using reference standards, but Metabolon is confident in their identities based on exact mass and fragmentation pattern.

**Figure 4:**
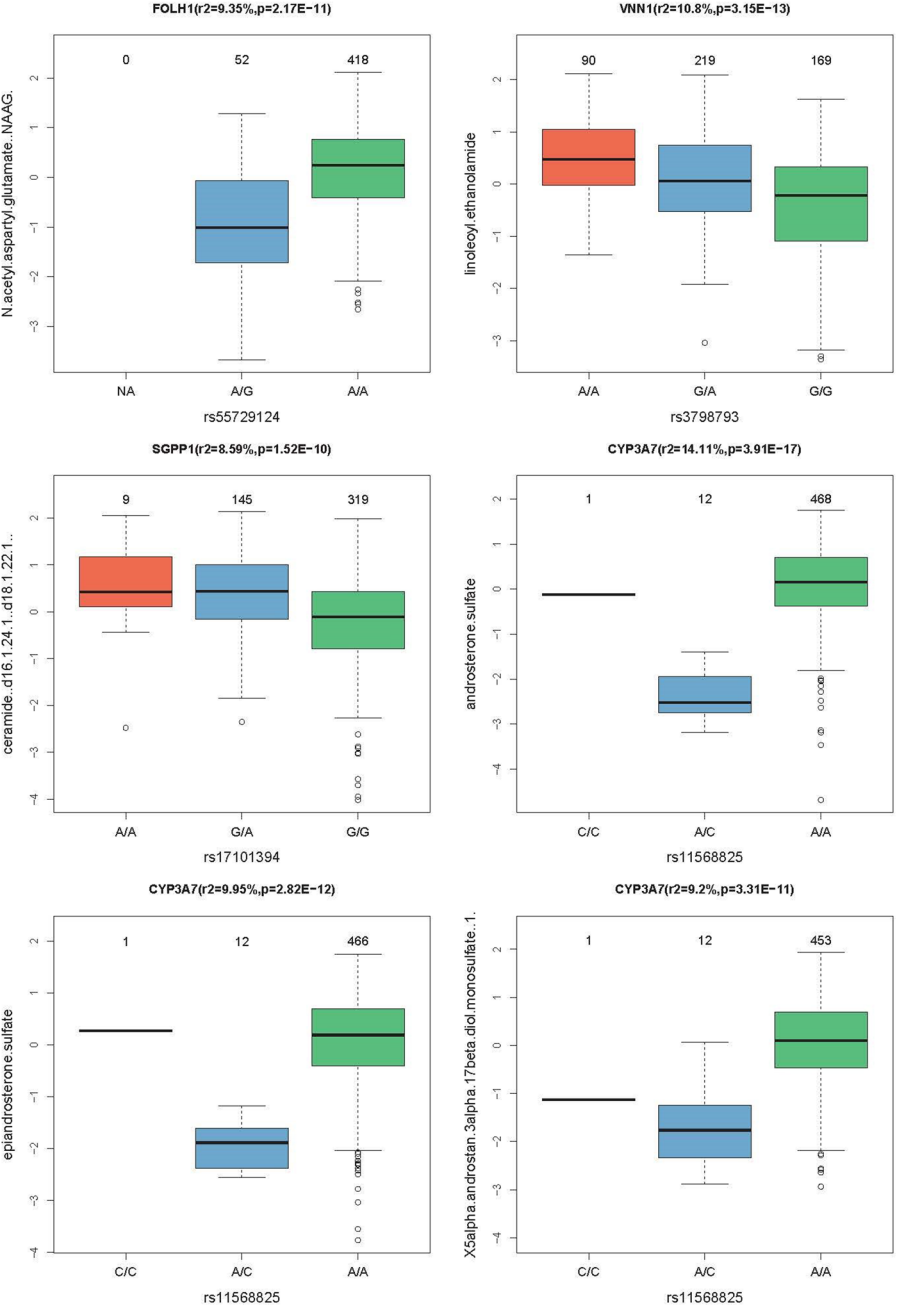
Boxplots of levels of metabolites by genotype for novel loci. Boxplots for the loci CYP3A7, SGPP1, VNN1, and FOLH1 indicating the metabolite level and the number of samples for each genotype group.

The 19 independent loci replicated 15 previously reported loci^26,27^ (Table 1). The remaining four mGWAS loci represented novel associations between specific SNPs and metabolites. Two of these included novel gene/metabolite associations, namely rs55729124 in folate hydrolase 1 (FOLH1) in association with N-acetyl-aspartyl-glutamate (NAAG) (r^2^ = 9.35%, p = 2.17E-11) and rs3798793 in vascular non-inflammatory molecule 1 (VNN1) in association with linoleoyl ethanolamide (r^2^ = 10.8%, p = 3.15E-13) (Table 1, Figs. 2 and 3). Other novel mGWAS included known gene/metabolite associations, but with novel SNPs. These included the third novel mGWAS rs11568825 SNP within the cytochrome P450 family 3 subfamily A member 7 (CYP3A7) gene, exhibiting significant association with three different metabolites (androsterone sulfate (r^2^ = 14.11%, p = 3.91E-17), epiandrosterone sulfate (r^2^ = 9.95%, p = 2.82E-12) and 5 alpha-androstan-3alpha, 17 beta-diol monosulfate 1 (r^2^ = 9.2%, p = 3.31E-11)). The fourth novel mGWAS represented association between rs17101394 in sphingosine-1-phosphate phosphatase 1 (SGPP1) gene and Cermamide (r^2^ = 8.59%, p = 1.52E-10). For the 4 novel mGWAS loci, there were clear genotype-dependent effects on levels of associated metabolites as shown in boxplots in Fig. 4.

**Figure 5:**
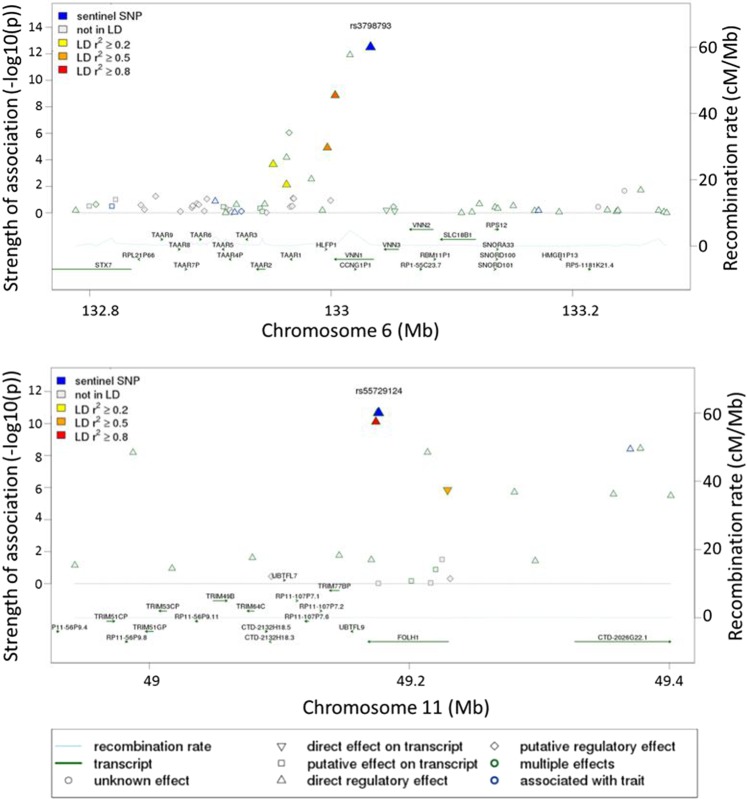
Regional association plots for the two new loci (VNN1 and FOLH1).

Regional association plots for the novel loci VNN1 and FOLH1 are shown in Fig. 5. The intronic SNPs within VNN1 (rs3798793, Fig. 5a) and FOLH1 (rs55729124, Fig. 5b) loci show the strongest association (−log10 (p-value)) with linoleoyl ethanolamide and N-acetyl-aspartyl-glutamate (NAAG), respectively. The colors correspond to different linkage disequilibrium (LD) thresholds, where LD is computed between the sentinel SNP (lowest p-value, colored in blue) and all SNPs.

### Common variant loci influence metabolites (mQTLs) in elite endurance athletes

**Figure 6:**
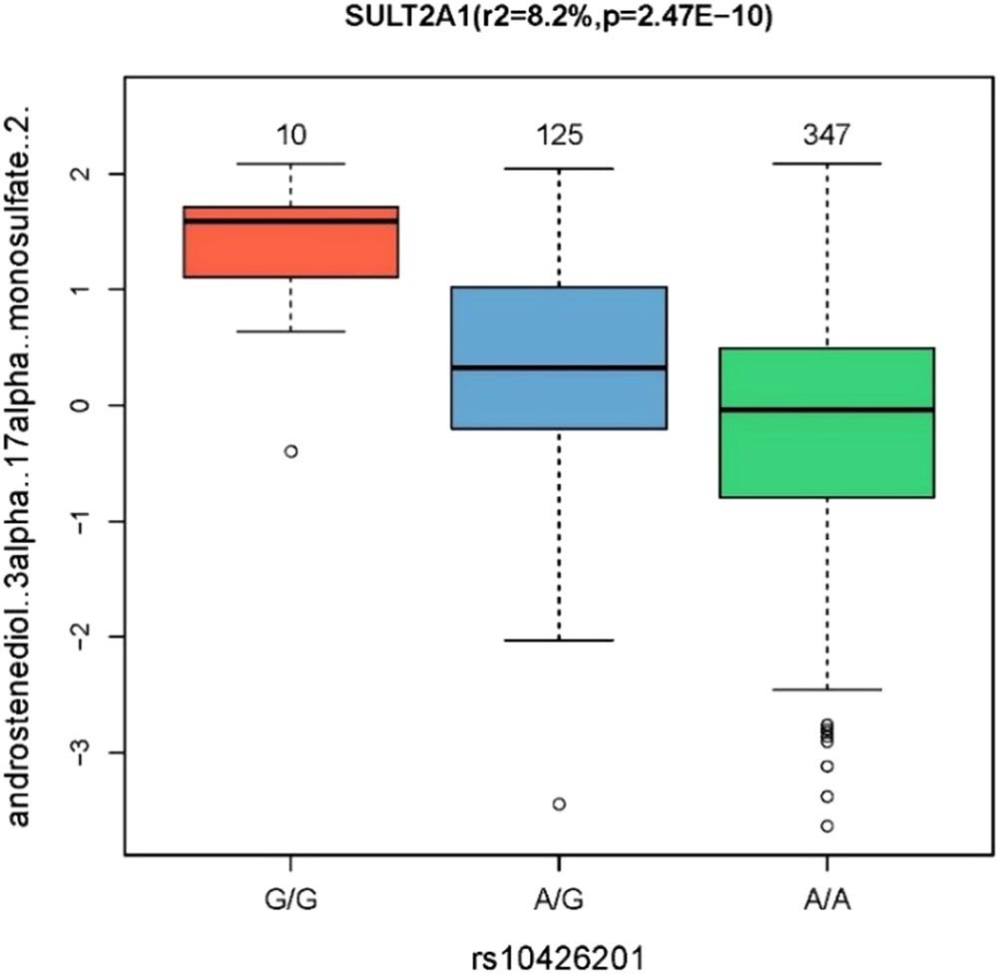
Boxplot for metabolite-locus pair associated with endurance, indicating the metabolite level and the number of samples for each genotype group.

In order to investigate novel mQTLs between athletes’ genotyping data and the confirmed 104 endurance metabolites (Table S3), significant (p < 0.05/104*275016 = 1.7 × 10^−9^) mGWAS associations were identified from amongst the list of significant mGWAS hits at p value < 10^−6^ (Table S4). Four significant associations were found including one novel mGWAS association between rs10426201 in SULT2A1 gene and androstenediol (3alpha, 17alpha) monosulfate (2). Although the latter association was reported before, it did not reach statistical significance^27^ (Table 2). For the novel endurance mGWAS locus in SULT2A1 gene, there was a clear genotype-dependent effect on levels of associated metabolite as shown in Fig. 6.

**Table 2:** Unique locus-metabolite pairs associated with endurance sports in comparison with previous reports, including one novel association between a known locus (SULT2A1) and a new metabolite.

Elite athletes											Non-elite athletes^27^	
Gene	rsID	Chr	Position	Function GVS	N	Beta	SE. Beta	P. Value	Metabolite	SUB_PATHWAY	Metabolite	P. Value
SULT2A1	rs10426201	19	48384749	intron	470	0.52	0.08	2.47E-10	androstenediol (3alpha, 17alpha) monosulfate (2)	Androgenic Steroids	4-androsten-3alpha,17alpha-diol monosulfate (2)	2.70E-07
SLC22A16	rs12210538	6	110760008	missense	457	−0.47	0.07	5.19E-10	dihomo-linoleoylcarnitine (C20:2)*	Fatty Acid Metabolism(Acyl Carnitine)	linoleoylcarnitine	1.58E-24
SLC22A24	rs75859219	11	62913676	upstream-gene	462	0.96	0.13	5.04E-13	etiocholanolone glucuronide	Androgenic Steroids	etiocholanolone glucuronide	9.13E-38
CYP3A7	rs45446698	7	99332948	upstream-gene	468	1.54	0.20	3.07E-13	16a-hydroxy DHEA 3-sulfate	Androgenic Steroids	16a-hydroxy DHEA 3-sulfate	2.07E-47

**Figure 7:**
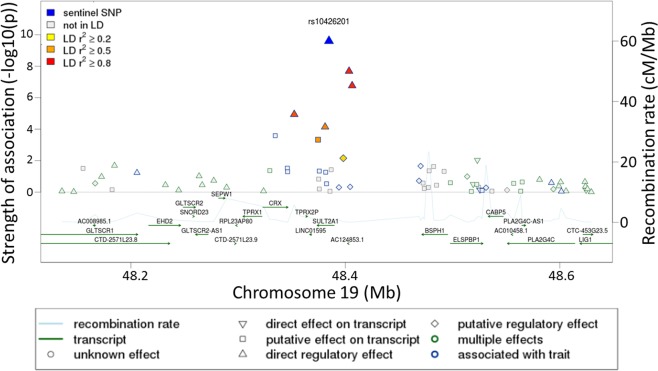
Regional association plots for the novel locus SULT2A1.

The regional association plot indicates that the intronic SNP (rs10426201) in SULT2A1 gene shows the strongest association (−log10 (p-value)) with androstenediol (3alpha, 17alpha) monosulfate (2) (r2 = 8.2%, p = 2.47E-10) (Fig. 7). The colors correspond to different LD thresholds, where LD is computed between the sentinel SNP (lowest p-value, colored in blue) and all SNPs.

## Discussion

Historically, the superior performance of elite athletes has been considered an outcome of a special talent shaped by intensive training^28^. The talent is currently believed to be a product of additive genetic components predisposing elite athletes to higher endurance/power trainability under the control of strong environmental cues including exercise and nutrition^29^. Despite the identification of a number of genetic variants associated with athletic endurance, their small effect size made it difficult to replicate in various small cohorts of elite athletes. Therefore, this study aimed to identify intermediate phenotypes (metabolites) that could offer direct functional relationship with genetic variants in elite athletes in response to their unique environment, hence provide a greater effect size and a better chance to be identified. This could help in understanding the superior physical and mental performance of professional elite athletes and the identification of novel exercise-related biomarkers in athletic candidates.

To achieve our aims, genotyping was performed in elite athletes from different sport disciplines using a druggable genes-enriched SNP chip. This chip covers various metabolic pathways suited for the investigation of our intermediate phenotype of interest (metabolites) without enduring the penalty of multiple testing associated with more comprehensive SNP chips^30^. This was followed by serum metabolomics of the same samples to confirm our previously published endurance metabolites^12^ using Metabolon platforms that offer a very comprehensive untargeted metabolomics profiling^31^. Subsequent mGWAS analysis was performed to reveal novel SNP-metabolite associations by comparing mGWAS hits identified in elite athletes with the reference studies that were previously performed in non-elite athletes^25–27^. Finally, novel mGWAS hits associated with endurance sports were determined using the confirmed list of endurance metabolites from the meta-analysis.

Our genotyping data revealed a number of SNPs associated with endurance but none has reached the GWAS level of significance (data not shown). This expected outcome may have resulted from our small sample size, small effect size of genetic variants and the complex phenotype of physical performance. Therefore, a more precise phenotype (metabolites) was sought to obtain larger effect size and a better chance for detection. In our pilot study of 191 elite athletes we identified a number of metabolites associated with endurance^12^. In this study, we confirmed a number of these metabolites by carrying out metabolomics in a second cohort, followed by a meta-analysis of the two cohorts. Among confirmed hits, elevation of pregnenolone and androgenic steroids indicate active steroid biosynthesis pathway in high endurance athletes. Reduced diacylglycerols and acyl carnitines and increased monohydroxy fatty acids suggest active fatty acid oxidation for energy generation in the high endurance group. Reduction in gamma glutamyl amino acids and glutathione metabolism suggests active oxidative scavenging mechanisms in moderate endurance group. These metabolic changes seen in high performance elite athletes may reflect various cellular adaptations to prolonged exercise-induced oxidative stress. These may include modulation of energy utilization, muscle mass and deployment of stress-scavenging mechanisms as previously suggested^12^.

Following genotyping and metabolomics analyses, genetically-influenced metabolites were firstly sought between elite athletes cohort and published data from non-elite athletes^25–27^, and secondly within the elite athletes cohort between moderate and high endurance groups. Both analyzes revealed novel mGWAS associations with significant effect size (between 8–14%, Table 2 and Fig. 3), clear genotype-dependent effect (Figs. 4 and 6) and evidence of multiple SNP associations within the same genomic region (Figs. 5 and 7).

The mGWAS results between elite and non-elite athletes revealed 4 novel mQTLs. The first of which is a negative association between rs55729124 in Folate Hydrolase 1 (FOLH1) and NAAG levels. FOLH1 encodes a type II transmembrane glycoprotein termed glutamate carboxypeptidase II (GCPII) that hydrolyzes NAAG to NAA and glutamate^32^. The intronic SNP rs55729124 may therefore be associated with enhanced FOLH1 activity leading to the breakdown of NAAG and accumulation of NAA and glutamate. NAA is a nervous system specific metabolite found predominantly in cell bodies of neurons. Aerobic fitness was reported to increase NAA levels, leading to improved cognitive enhancement^33^. The identification of this novel mQTL in elite athletes may suggest augmentation of FOLH1 activity in elite athletes with exercise, resulting in higher NAA levels compared to non-elite athletes in other published studies (Table 3). Interestingly, NAAG serves as a reservoir to provide glutamate to cancer cells through GCPII^34^. The identification of this novel mQTL could potentially be utilized for the development of novel strategies for targeting GCPII for cancer treatment.

**Table 3:** Novel elite athletes-associated mQTLSs reflecting gene/environment (exercise) interaction.

SNP	Gene	Metabolite	Functional relationship between gene and metabolite	Interaction with athletic performance (exercise)
rs55729124	FOLH1	N-Acetylaspartylglutamic acid (NAAG)	Gene encodes an enzyme that directly cleaves NAAG into NAA + Glutamate	Aerobic fitness was reported to enhance NAA levels, leading to increased cognitive enhancement^33^
rs3798793	VNN1	Linoleoyl ethanolamide	Gene encodes a membrane protein that participates in hematopoietic cell trafficking	Exercise increases serum concentrations of endocannabinoids including linoleoyl ethanolamide^36^
rs17101394	SGPP1	Ceramide	Gene encodes enzyme that directly mediates recycling of sphinogsine into cermides	Acute prolonged exercise was shown to influence ceramide metabolism in human skeletal muscle^42^

The second mQTL identified in our athletic cohort is a positive association between rs3798793 in vascular non-inflammatory molecule 1 (VNN1) in association with linoleoyl ethanolamide. VNN1 protein possess pantetheinase activity that may play a role in oxidative-stress response. The endocannabinoid linoleoyl ethanolamide has a role as fatty acid amide hydrolase inhibitor as it inhibits arachidonoylethanolamide amidohydrolase. It has also been shown to have a neuroprotective role during ischemia reperfusion injury with potential therapeutic benefits when used as complementary treatment with other therapies to improve stroke outcome^35^. The link between VNN1 and linoleoyl ethanolamide is not clear yet. Exercise, however, has been shown to increase serum concentrations of endocannabinoids^36^, thus the identification of this novel mQTL may be due to exercise interaction (Table 3).

The third mQTL involved association of various SNPs in the Cytochrome P450 Family 3 Subfamily A Member 7 (CYP3A7) with lower serum sulfated steroids^37^. This is the first report of a negative association between rs11568825 in CYP3A7 with 5alpha-androstan-3alpha,17beta-diol monosulfate (1), although association of other SNPs within the same gene with same metabolites were previously reported^27^. Cytochrome P450 enzymes are important for the metabolism of many endogenous compounds including various steroids^38^. We have shown previously that 5alpha-androstan-3alpha,17beta-diol monosulfate is increased in endurance sports, potentially providing evidence of environmental interaction with endurance exercise^12^ (Table 3). Previous studies have reported that signaling activated by 5alpha-androstane-3alpha,17beta-diol may represent a novel pathway responsible for the progression to androgen-independent prostate cancer^39^. Therefore, the identification of this novel mQTL may potentially aid in designing novel therapeutic targets for androgen-independent prostate cancer.

The fourth novel mQTL was a positive association between rs17101394 in Sphingosine-1-Phosphate Phosphatase 1 (SGPP1) in association with Ceramide (d16:1/24:1, d18:1/22:1). Although the association of the same SNP with multiple different metabolites was previously reported. These metabolites included various spingolipids such as palmitoyl dihydrosphingomyelin (d18:0/16:0), sphingomyelin (d18:1/14:0, d16:1/16:0), sphingomyelin (d18:1/15:0, d16:1/17:0), sphingomyelin (d18:1/20:0, d16:1/22:0), and sphingomyelin (d18:1/21:0, d17:1/22:0, d16:1/23:0)^27,40^ as well as X-08402, and X-10510^26^ that are also related to sphingolipid pathway^20^. SGPP1 catalyzes the degradation of Sphingosine-1-phosphate (S1P), a bioactive sphingolipid metabolite that regulates diverse biologic processes, via salvage and recycling of sphingosine into long-chain ceramides^41^. Acute prolonged exercise was shown previously to influence ceramide metabolism in human skeletal muscle^42^, perhaps explaining identification of this mQTL in our elite athlete cohort (Table 3). Additionally, the identification of this mQTL could potentially be utilized for the development of novel therapeutic strategies against atherosclerosis since sphingolipids have been directly related to increased risk of atherosclerosis^43^.

In addition to novel mQTLs identified in elite athletes, we have confirmed 16 previously published mQTLS, two of which exhibited greater percent of variance in our elite athletes compared to the ones reported in non-elite athletes^25,27^, including rs7072216 in PYROXD2 in association with N-methylpipecolate (effect size 43% vs 31%) and rs1881245 in NAT8 in association with N-acetyl-1-methylhistidine (effect size 30% vs 26.6%). Among the 16 previously reported loci, 9 loci showed similar direction of association compared to previously published studies^25,27^. Among these, 4 loci showed more than 2 fold increase in their effect size in elite athletes including CYP3A7, AGMAT, NAT8 and SPTLC3 in association with androsterone sulfate, beta-guanidinopropanoate, N-acetyl-1-methylhistidine, and sphingomyelin (d18:1/25:0, d19:0/24:1, d20:1/23:0, d19:1/24:0). Whereas 6 loci showed opposite direction of association compared to previously reported study including CERS4, KLKB1, SLC6A13, SLCO1B1, TMPRSS11E and UGT1A10^27^. Among these, SLCO1B1 locus showed 2.8 fold decrease in its effect size in association with glycochenodeoxycholate glucuronide (1) in elite athletes. The functional relevance of these variable effect sizes remains to be investigated.

When focusing on confirmed endurance metabolites, four mGWAS were identified, of which a positive association between rs10426201 in Sulfotransferase Family 2 A Member 1 (SULT2A1) in association with androstenediol (3alpha, 17alpha) monosulfate (2) was novel. SULT2A1 catalyzes the sulfation of steroids, a process that is fundamental for their function. Following biosynthesis, hydrophobic steroids become sulfated to accelerate their circulatory shuttling to target tissues. The expression of anion transporting polypeptides on target cells enables their uptake. Subsequently, intracellular sulfatases activate them by hydrolyzing the steroid sulfate esters^44^. The genetic predisposition of steroid sulfation in elite high endurance athletes may therefore explain active steroid biosynthesis in this group, and could potentially contribute to their elite physical performance. Furthermore, the identification of the genetic predisposition to enhanced activity of SULT2A1 could potentially be utilized to determine the percentage of sulfated intact molecules with relevance to steroid profiling parameters for antidoping strategies^45^.

Study limitation: The use of Mitchell’s criteria based on sport disciplines to dichotomize participants into two endurance groups^12,46^ is a crude method of categorization. A better phenotype would be the actual measurement of VO2max in these athletes. However, due to the strict institutional research board’s instructions, the only available information about participants were their sport disciplines and gender. Additionally, the relatively small number of participants may have limited the power of the study, however this remains the largest cohort of elite athletes with mGWAS data to date as elite athletes samples are very difficult to obtain. Future studies are warranted to confirm these findings in larger cohorts using more accurate measures of endurance.

This study reveals for the first-time evidence of genetically-influenced metabolites associated with elite athletic status in general and endurance sports in particular. Uncovering these novel associations in elite athletes, but not in the general population, could reflect a gene-environment (intensive exercise) interaction that augments the effect size of these genetic variants. Among the novel identified mQTLs, SNPs associated with enhanced endogenous steroids activity may play an important role in elite athletic performance, especially among endurance athletes. The utilization of these mQLTs as biomarkers for selecting athletic candidates with a greater potential to becoming elite endurance athletes is warranted and should be further validated. Additionally, the newly identified mQTLs in elite athletes could provide crucial information about the interaction between exercise and genetic predisposition of some doping-related metabolites, potentially paving the way for development of non-traditional indirect analytical strategies for the detection of novel doping strategies. Finally, the identification of these novel mQTLs could provide vital clues for potential therapeutic targets as they provide direct functional relationships between genes and their products/byproducts with therapeutic values.

## Subjects and Methods

### Cohort

Blood and serum samples were collected at anti-doping laboratories in Qatar (ADLQ) and Italy (FMSI) from 490 elite athletes who participated in national or international sports events and tested negative for doping abuse. Written informed consent was obtained from each participant. This study was performed in line with the World Medical Association Declaration of Helsinki – Ethical Principles for Medical Research Involving Human Subjects. All protocols were approved by the Institutional Research Board of ADLQ (F2014000009). Table S1 summarizes the distribution of all recruited athletes according to their sports disciplines into moderate and high endurance, groups following published criteria^46^. It was not possible to involve patients or the public in this work.

### Metabolomics

Profiling of serum metabolites in 490 elite athletes (Table S1) was performed using protocols established at Metabolon, Durham, NC, USA. The platform utilizes Waters ACQUITY ultra-performance liquid chromatography (UPLC) and a Thermo Scientific Q-Exactive high resolution/accurate mass spectrometer interfaced with a heated electrospray ionization (HESI-II) source and Orbitrap mass analyzer operated at 35,000 mass resolution. Detailed protocol and QC measures were previously published^12,31^.

### Genotyping methods

Genotyping of 490 elite athletes was conducted using Illumina Drug core BeadChip arrays. The chip contains 476728 SNPs including 240,000 highly-informative genome-wide tag SNPs and a novel 200,000 custom marker set designed to support studies of drug target validation and treatment response. The latter SNP set was selected to include the following: 1- genes involved in drug absorption, distribution, metabolism and excretion (ADME), 2- exome content coverage of genes encoding proteins closely related to targets of approved small molecule and biotherapeutic drugs or binding drug-like compounds, and 3- other useful content, including all SNPs associated at GWAS significance with any human trait marking the X and Y chromosomes and mitochondrial DNA, and for sample fingerprinting (common SNPs represented on major genome-wide array products from both Illumina and Affymetrix). These SNPs are expected to represent genes involved in controlling the same essential metabolic pathways that regulate the magnitude of physical performance. Following genotyping using Illumina’s Drug Core SNP array, the following SNP exclusion QC filters were adopted: genotype call rate < 98% (130526 SNPs were excluded), MAF < 0.01 (70210 SNPs were excluded) and Hardy Weinberg p value < 10^−6^ (976 SNPs were excluded), resulting in 275016 SNPs (Bonferroni significance = (0.05/(275016 × 751) = 2.4E-10) used for the analysis. Genotype distribution was compared among athletes grouped according to the endurance group of their respective sports (data not shown).

### Statistical analysis of metabolomics data

A linear regression model was run using R statistical package (version 2.14, www.r-project.org/) to assess association between metabolites and endurance level (moderate versus high). The model also corrected for the following possible confounders: sport power, gender, hemolysis levels (determined visually by Metabolon) and metabolites PCs. Multiple testing was Bonferroni corrected. A meta-analysis was utilized to identify metabolites equally influenced by endurance level in both metabolomics datasets in the current study and previously published study^12^. Initially, functions from the R library ‘esc’ were used to convert the beta value from the regression analysis of individual datasets into effect size (in this case, difference in mean between low and high levels of endurance). The metafor R library was then used to run the metanalysis on the derived effects size from the individual datasets. The p-values from the meta-analysis were corrected for multiple testing based on FDR correction.

### mGWAS analysis

Associations between SNPs and metabolite levels were computed using lm function in R (version 3.3.1) while correcting for gender, hemolysis and predicted ethnicity based on caparison with 1000 genome project that was calculated with plink version 1.9. An additive inheritance model was used (SNPs were coded as 0,1,2 according to their genotype group). Manhattan and box plots were generated using R (version 3.3.1). Regional association plots were produced using SNIPA (http://snipa.helmholtz-muenchen.de/snipa/). Loci and sentinel SNPs association results were divided into gene loci, and in each of these the sentinel SNP and sentinel metabolite were defined with 500Kb according to the SNP-metabolite association with the highest significance, defining the metabolite quantitative trait loci (mQTLs). In the case where a locus had sentinel SNP that could not be found in regional association plotting release grch37-1kgpp3v5 (eur) and lying in location of known gene, it was mentioned in association with known SNP loci. For example, SNP rs3733402 in locus 5 had P-value of 6.80E-12, however in regional association rs4241816 was indicated as it had the 2nd highest p-value 2.02E-11 in locus 5. Both are associated with same gene KLKB1.

### mQTLS associated with Endurance

To determine mGWAS associated with endurance sports, SNPs that were significantly associated with 104 endurance metabolites (Table S2) were identified within the list of mQTLs from the mGWAS analysis (Table S4). Bonferroni p-value of 1.7 × 10^−9^ [0.05/(104 × 275016)] was used to report a significant association.

### Ethics approval and consent to participate

This study was performed in accordance with the World Medical Association Declaration of Helsinki. All protocols were approved by the Institutional Research Board of anti-doping lab Qatar (F2014000009) and participants have given consent to participate.

## Supplementary information

Supplementary information

Supplementary Dataset 1

## Data Availability

All relevant data are within the manuscript and its Supporting Information files.
