# Author Correction: Metabolic GWAS of elite athletes reveals novel genetically-influenced metabolites associated with athletic performance

**DOI:** 10.1038/s41598-020-67555-9

**Published:** 2020-06-23

**Authors:** Fatima Al-Khelaifi, Ilhame Diboun, Francesco Donati, Francesco Botrè, David Abraham, Aroon Hingorani, Omar Albagha, Costas Georgakopoulos, Karsten Suhre, Noha A. Yousri, Mohamed A. Elrayess

**Affiliations:** 1 Anti Doping Laboratory Qatar, Sports City, Doha, Qatar; 2 Division of Medicine, University College London, London, NW3 2PF UK; 3 College of Health and Life Sciences, Hamad Bin Khalifa University, Doha, Qatar; 4 Laboratorio Antidoping, Federazione Medico Sportiva Italiana, Largo Giulio Onesti 1, 00197 Rome, Italy; 5 UCL Institute of Cardiovascular Science, University College London, London, WC1E 6BT UK; 6 Center for Genomic and Experimental Medicine, University of Edinburgh, Edinburgh, UK; 7 Department of Physiology and Biophysics, Weill Cornell Medical College in Qatar, P.O. Box 24144, Qatar-FoundationDoha, Qatar; 8 Department of Genetic Medicine, Weill Cornell Medical College in Qatar, P.O. Box 24144, Qatar-FoundationDoha, Qatar; 9 Computer and Systems Engineering, Alexandria University, Alexandria, Egypt; 10 Biomedical Research Center, Qatar University, Doha, Qatar

Correction to: *Scientific Reports* 10.1038/s41598-019-56496-7, published online 27 December 2019

The Acknowledgements section in this Article is incomplete.

“This study was funded by Qatar National Research Fund (QNRF), Grant number NPRP7-272-1-041 (MAE, KS, CG and FB). The funders had no role in study design, data collection and analysis, decision to publish, or preparation of the manuscript.”

should read:

“This study was funded by Qatar National Research Fund (QNRF), Grant number NPRP7-272-1-041 (MAE, KS, CG and FB). The funders had no role in study design, data collection and analysis, decision to publish, or preparation of the manuscript. The publication of this article was funded by the Qatar National Library.”

