# Supplementary material for: Metabolic GWAS of elite athletes reveals novel genetically-influenced metabolites associated with athletic performance
Source: Sci Rep. 2019 Dec 27;9:19889. doi: 10.1038/s41598-019-56496-7 (PMC6934758; doi:10.1038/s41598-019-56496-7)
Supplement: Supplementary file 1 — Supplementary information [file 41598_2019_56496_MOESM1_ESM.pdf]

Metabolic GWAS of elite athletes reveals novel genetically-influenced metabolites associated with athletic performance

Fatima Al-Khelaifi, Ilhame Diboun, Francesco Donati, Francesco Botrè, David Abraham, Aroon Hingorani, Omar Albagha, Costas Georgakopoulos, Karsten Suhre, Noha A. Yousri, Mohamed A Elrayess

Supplementary table legends

Table S1. Distribution of recruited athletes according to their sports disciplines (M: Male, F: Female)

Table S2. List of metabolites identified by Meta-analysis in current and prevoiusly published cohort in association with endurance sports

Table S3. List of common variant loci influence metabolites (mQTLs) in elite athletes

Table S4. List of significant mGWAS hits at p value < 10^-6
